# Shakers and Shakerism

**Published:** 1887-11

**Authors:** Hester M. Poole


### SHAKERS AND SHAKERISM.

BY HESTER M. POOLE.

A late visit to the Shakers at the instance of one of their elders, filled me with a desire to lay before your readers some account of these people so interesting to the thoughtful student of humanity, yet so little understood.

On the eastern boundary of the State of New York, twenty miles as the crow flies from the Hudson, and contiguous to the beautiful hills of Berkshire, in Massachusetts, lies about six thousand acres of land owned and tilled by the Shakers of Mt. Lebanon.

A lovely and peaceful scene expands before the visitor who rides through these well-tilled farms and inspects workshops and dwellings. Along the street one group of buildings succeed another, five in all, containing three hundred or more of both sexes and all ages. Each group constitutes a family, presided over by two men and two women, whose wisdom, patience, and tenderness are constantly challenged in administering more especially to the spiritual necessities of those under their charge. They are assisted in the temporal affairs by two deacons aud two deaconesses, whose wisdom is available in all matters pertaining to the good of the society.

The family life is that of a religious communism, the intention being as far as possible, to preserve and perpetuate primitive Christianity. Body and soul are consecrated to this purpose. It is a part of their unwritten creed to study the laws of hygiene and conform to them, to live in celibacy, and to exercise justice in the earning, owning and distribution of property.

Among them are neither bond nor free, rich nor poor. All are incited to industry, thrift, generosity and fraternity, and there is a strong psychologic power in such sentiments, which, when exercised by masses of people, produces an influence that not even the stranger within the gate can quite escape. The despot or the millionaire would feel out of place among those "gentle ascetics," whose lives are a rebuke to that spirit of greed, selfishness and love of luxury which is the curse of modern civilization. We find at Mt. Lebanon several hundred people living in a simple, pure, wholesome manner, without the help of courthouse, jail, grog shops or the three professions, so that even from an external point of view, Shakerism is eminently successful.

All the buildings occupied by the respective families, constructed of wood, brick and stone, are commodious and well ventilated. The arrangements for cooking and eating are admirable; in fact, in regard to appliances for comfort and sanitation they take the lead among progressive peoples.

The table, almost entirely vegetarian, is perfect. Food is fresh, abundant, exquisitely cooked and served with care and intelligence. Cereals with the exception of superfine flour, are cleansed and crushed in their own mills and used in a variety of ways. There is a large dairy, and tons of fruit deliciously prepared, are ranged in storerooms for the winter's consumption. Woman's work is simplified by curious machinery invented and made by some of their leaders. All work, but none overwork. Garments are homemade and until lately woolen clothing was homespun and home-woven. An abundance of spring water is carried into every building, ventilation and drainage are excellent and sickness is almost a myth. Cleanliness of the person and of their dwellings is carried to its utmost extent. It follows that simplicity of furnishing is necessary, and that their apartments, in comparison with those of the world, look plain and bleak.

Yet recreation and rest, sunshine and cheerfulness are terms having real meaning. "Age cannot stale nor custom wither" men and women who live so near to nature and in the exercise of such noble qualities. Accordingly they very generally appear to be from ten to twenty years younger than they really are. Many reach extreme old age and finally pass away from the natural decay of the body, with little sickness or pain. The expression of the face is mild, benignant and serene, sometimes approaching high spiritual beauty.

So much for the religion of the body—the only basis of the scientific and enduring.

Before reviewing their religious tenets it may be well to state that their origin is found in the Revolutionists of Dauphine and Nivarais, France, about the year 1689. Offshoots of the parent stock formed a society in England in 1747; and two years prior to the Declaration of Independence by the American colonies, Ann Lee, with seven of her followers, landed on these shores.

From the little spark brought over by them a fire was kindled which vivified many souls, and in New Lebanon over a century ago, these gathered together and built their first house for public worship. From that period they have acted as a leaven among the elements of progress.

Mother Ann, so-called from that tender maternal love which would fain save a world from sin and suffering, was the first seer to enunciate the principle that the Great First Cause is dual—He and She—Father and Mother. It is certain that Theodore Parker obtained his conception of this deific attribute from the Shakers, as shown by his correspondence. This duality is now so generally accepted that churchmen are apt to forget that the Jewish Jehovah and the Christian God was forceful, revengeful and on occasion hateful. This one-sided Creator lacked all that sweet plentitude of womanly love, which united with a manhood of corresponding wisdom, would alone be worthy of reverence And Christendom waited seventeen centuries for a woman to declare the duality of the Deific Essence.

This, then, is the central idea of Shakerism. Ranged about it are others, not the result of dry reasoning, but of experiences similar to those of Paul and the Pentacostal church. Profoundly reverent by nature they recognize a "divine afflatus," which is the inspiration of all real development. This divine element they believe has manifested itself whenever the condition of an individual or of society afforded occasion, from the beginning of history through Moses, Isiaah, Swedenborg, Whitfield and others down to the time of Mother Ann, and even since then. They declare that "the continuous revelations of truth will ever be the leading lines of human progress."

What is now known as modern Spiritualism is accepted by them as a fact. They assert that all phases of mediumship were common among them several years prior to the first rap heard at Hydeville and that its advent to the general public was then foretold. In its higher phases, shorn of crudities and monstrosities, it is still sometimes exhibited. Witness the sweet, pathetic yet simple melodies which come, "the gift of the spirit," as they believe, to one another, either in private or in public worship. A brother or a sister at such times is inspired to sing a new song to new music, which, when written down becomes a permanent possession. A large book has been published consisting of these inspirational hymns, which is in constant use.

They do not generally believe in the miraculous birth or divinity of Jesus, but consider that he was divine in the sense of having power to rise above the lower propensities. His mission was "simply and fully to manifest the divine attributes to man" more than any other one who has ever lived.

They also believe that the first wave of deific light sweeping over the earth after the Reformation, began with the Quakers. Its mission was to "prepare the world for the divine form of human society," or the "kingdom of heaven on earth." The second appearance of this wave or the "Christ-Spirit" was manifested in and through woman in the form of Ann Lee.

They accept the Christian Bible allegorically and literally, and include among Bibles, the Koran, Talmud, Zendavesta and other books sacred to various nations. They discountenance war, never go to law among themselves, and aim to act in a just, humane and brotherly manner to all men.

In regard to women "it is the only society in the world, so far as we know," said Eldress Anna, "where woman has absolutely the same freedom and power as man in every respect." And the world may well hail the advent of woman's era if it shall usher in such noble types of womanhood as we found at Mt. Lebanon, hid under the quaint cap and staid dress of the gentle sisterhood.

In regard to the future, Elder Evans has declared their belief to be that "The old heavens and earth—united church and state—are fast passing away, dissolving with the fire of spiritual truth. Out of the material of the old earthly, civil governments, a civil government will arise—is even now arising—in which right, not might, will predominate. It will be purely secular, a genuine Republic. Men and women will be citizens. All citizens will be free-holders. They will inherit and possess the land by right of birth. War will cease with the end of the old monarchial, theological earth. * * * In the new earth sexuality will be used only for reproduction ; eating for strength, not gluttony ; drinking for thirst, not drunkenness. And property, being the product of honest toil—as those who will not work will not be allowed to eat—will be for the good of all, the young and old."

Purity of mind and body is necessary to Shakerism. But virgin celibacy has in it nothing of moroseness or asceticism. A pleasant relation is maintained between the brethren and sisters, fostered by social meetings in which reading, conversation and discussions upon topics germane to the welfare of humanity take place. In these, all who choose to do so, participate.

Believing that human theologies perish in the using, while the revelations of truth are continuous and progressive, they earnestly watch and wait for every sign of the domination of the spirit of truth and justice, over that of error and falsehood in the government or in social life. As to them, the fall of man consists in "disorderly relationships," and the serpent is the sensuous nature. They are strenuous in the advocacy of purity and temperance. And here it may be said that the institution of marriage is not condemned by the Shakers. All men, they consider, are bound to make the animal propensities tributary to their higher natures while marriage is a purely worldly institution. They are called to a higher order of life, to "come out of the world and be separate."

The following description of this growth from a lower to an upper plane, is from the pen of one of their number who wears his eighty odd years as a crown of wisdom and beauty.

"Allow me to assure you, scientific men, philosophers, doubters, and all interested, that whenever human spirits are in the right condition and are about to change from the animal emotional, to the divine emotional life, that there will be manifestations of intelligent spiritual affinities, forces, effusions of the divine spirit, producing extraordinary results as on the day of Pentecost. There will be deep conviction of sin, bodily agitations, gifts of tongues, curing diseases, discernment of spirits and striking with fear the hardened sinner and unbelieving opposer."

Whatever may be thought of their beliefs, the catholicity of thought evinced by their leaders, the comprehensive grasp of affairs, the judgment of the trend and comparative value of social, political and religious movements, the balancing of various reforms, the interest maintained in scientific discoveries and inventions, the depth and breadth of that love of humanity which dominates every motive, is something as surprising as it is delightful to the dispassionate visitor.

Prof. Richard T. Ely, of Johns Hopkins University, who sojourned at Mt. Lebanon for a few weeks, gives this testimony in regard to that visit: "The feeling grew upon me that I was in a social observatory, viewing as from another planet the buying and selling, the hurrying to and fro, the marrying and giving in marriage, the toil, the pleasure, the vanity, the oppression, the good and evil among men on earth."

There are seventeen communities of Shakers in this country, containing in all between four and five thousand individuals. These are situated in the States of New York, Massachusetts, Connecticut, New Hampshire, Maine, Ohio and Kentucky. Elder F. W. Evans, the able and venerable senior elder at Mt. Lebanon, has just returned from a visit to England, at the solicitation of sympathizers in Great Britian who desire to establish a community. Adherents are constantly joining them, though in the nature of things not in large numbers. Those who believe and work in unison with their aims, yet who remain without the fold, are more numerous. However this may be, these people who dispense with liquor and tobacco, who subsist on grains and fruits, and live near to the great heart of nature, practice as well as preach a temperance and a religion well worthy of respectful attention.—*The Open Court.*

